# The One Health approach: reinventing our past knowledge to provide a sustainable future

**DOI:** 10.1093/af/vfaf015

**Published:** 2025-10-14

**Authors:** Elsio A Wunder

**Affiliations:** Department of Pathobiology and Veterinary Science, University of Connecticut, Storrs, Connecticut, USA; Gonçalo Moniz Institute, Oswaldo Cruz Foundation, Brazilian Ministry of Health, Salvador, Brazil

**Keywords:** one health, one medicine, interconnectedness, sustainable, ecosystems

**Implications**

- Animal, human, plant, and environmental health are intrinsically linked and are interdependent. Acting on ONE affects ALL.
- Collaboration between all sectors of our society is essential to prevent diseases, improve health, and provide a sustainable future with a well-balanced ecosystem.
- There is a need to change the way we address the complexity of threats and risks we face currently. Traditional medicine and health approaches, either human, veterinary, or ecological, will not provide a comprehensive answer. The One Health approach recognizes the interconnectedness of all sectors and is a valuable tool to achieve broader success.
- The One Health approach is internationally recognized and already a global reality. However, it is essential to push this approach to the forefront of our activities to improve health. There is a need to advance the One Health approach into the curriculum of schools, colleges, and universities, while engaging community partners and professionals from all areas, from social to basic science, to work toward sustainable and efficient solutions for health.

## One Health—An Ancient Concept

For centuries, native and indigenous populations around the globe interacted with their surroundings as part of their daily live routines to hunt and harvest for nourishment, to celebrate, and to survive. They understood that their actions had major consequences on crops, animals, rivers, and forests. They lived in harmony with nature to find a balance for their own healthy and long lives. Then came maritime expansion, colonization, urbanization, globalization, and somewhere along the way we lost the respect and connection with nature. But this ideal and this concept of connection was engraved in our memory and present around us, it just needed to be rediscovered and properly explored.

The ancient Greek philosopher Hippocrates (460 BCE–367 BCE) believed that human health was dependent on the environment surrounding us (Pappas et al., 2008). During the 19th and 20th centuries, physician Rudolf Virchow and his student William Osler went further. Virchow coined the term “zoonosis” to describe diseases transmitted between animals and humans, and together with Osler, considered the father of veterinary pathology in North America, they brought to the mainstream the importance of zoonotic diseases. They advocated the idea that there is no line dividing animal and human health, publishing on the relation of animals to man and promoting comparative pathology (Gyles, 2016). During the 20th century, the One Medicine idea was further pushed by Dr. James Steele, a veterinarian who recognized the importance of good animal health for good public health. In 1947 he founded the Veterinary Public Health Division at the Centers for Disease Control and Prevention (CDC), a government agency in the US, introducing to the world the important role of animals in the epidemiology of zoonotic diseases (Gyles, 2016). Then, in 1964 another veterinarian, Dr. Calvin Schwabe, coined the term “One Medicine” in his book *Veterinary Medicine and Human Health*, where he strongly advocated that professionals in human and veterinary public health should interact and collaborate to address zoonotic disease threats (Gyles, 2016).

The beginning of the 21st century saw an evolution of the concept of One Medicine. A set of conferences established by the Wildlife Disease Association and the Society for Tropical Veterinary Medicine addressed the need to encourage collaboration among specialists to promote health beyond the focus of solely treating diseases. The severe acute respiratory syndrome epidemic in 2003 helped the final evolution of One Medicine into One Health, shifting from a human-animal disease concept to a more broad ecosystem health approach, one that recognizes that plants and environmental factors, and their health, are essential to the overall health of humans and animals (van Helden et al., 2013; Gyles, 2016).

## One Health—A “New” Concept

Modern concepts regarding health, like global health and planetary health, have a very anthropocentric attitude, directing the focus to human health almost exclusively. One Health takes on a more broad, integrated, and unifying approach to balance and optimize the health of people, animals, and ecosystems in a sustainable way. At its core, One Health acknowledges and accepts the connectiveness and interdependency between the health of humans, domestic and wild animals, plants, and the wider environment and ecosystems (Lancet, 2023). Furthermore, One Health considers the integrative effort of multiple disciplines and sectors working locally, nationally, and globally to achieve evidence-based and community-placed solutions to complex health challenges (Bayisenge et al., 2022).

**Figure 1.**
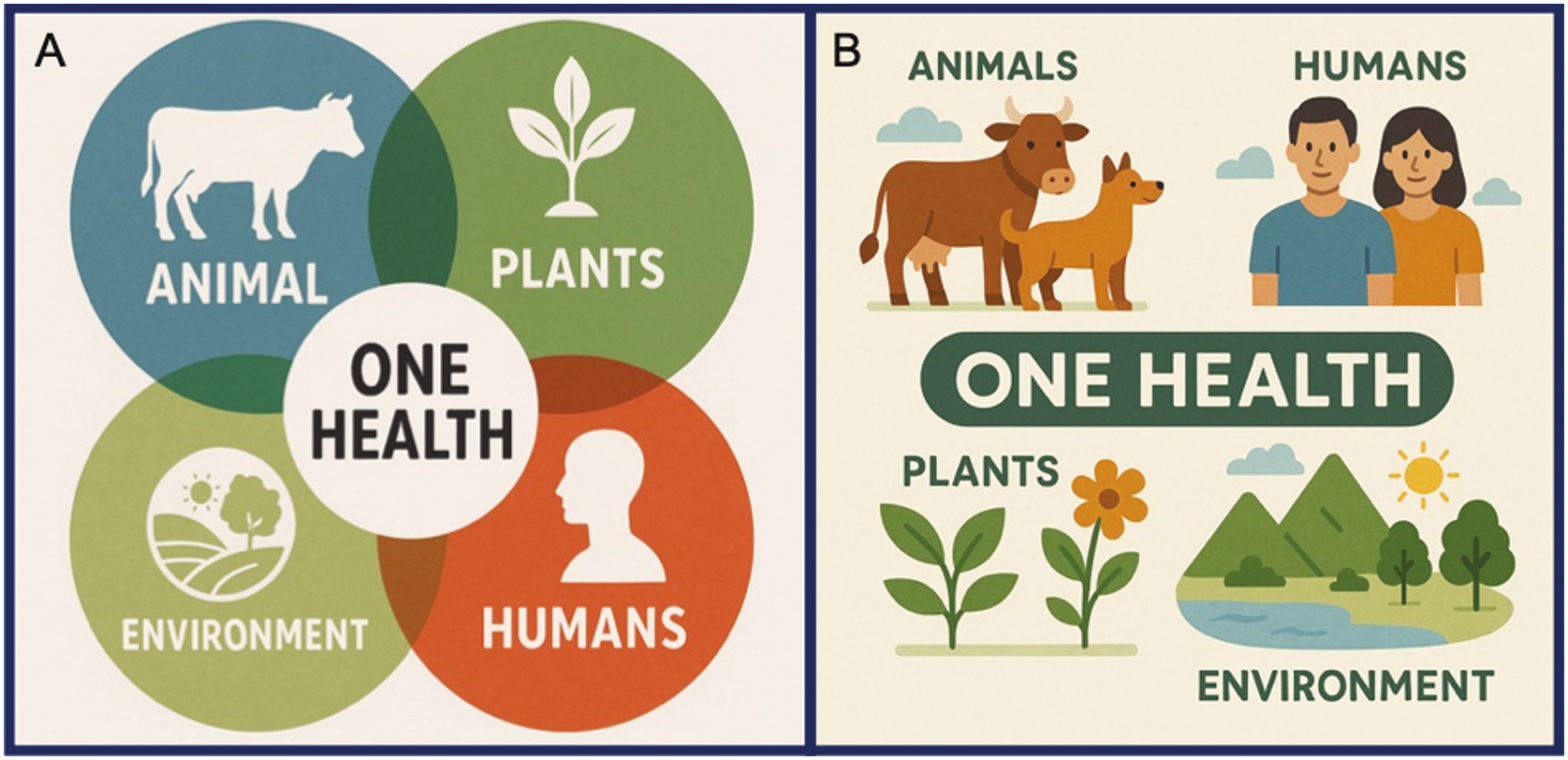
Examples of graphic representations of the One Health approach. Traditional representations of the interconnectedness of all sectors (A) and an optional example with a more integrated representation of all sectors (B) within One Health. Figures generated by AI.

One of the keywords for the One Health approach to take into consideration is interdependency. There is a false idea that One Health deals only with the area where humans, animals, plants, and the environment intersect. This is usually highlighted by the common graphic representation of the One Health approach showing intersected circles (Figure 1A). One Health does not deal exclusively with that small area of intersection between all sectors (Figure 1A), it assumes that humans, plants, animals, and the environment are ONE and each needs equal attention to ensure optimal health for all (van Helden et al., 2013). Ideally, a simpler graphic version would be a better and less misleading representation of the One Health concept (Figure 1B).

Since 2008, the One Health is a recommended approach and a political reality (FAO, 2008). Recently there have been successful examples of the use of the One Health approach in areas related to food safety, antimicrobial resistance, zoonotic diseases, and water and sanitation (Zinsstag et al., 2023). However, the lack of engagement and the limitations to bring forth this approach to all sectors has hampered the benefits that this approach can promote (van Helden et al., 2013).

## One Health—The Concept for the Future

Our world is rapidly changing around us and the consequences are extreme. Rapid urbanization and widening social inequality have led to the dramatic growth of disadvantaged low-income urban settlements in developing countries. Currently, one billion of the world’s population lives in low-income neighborhoods under precarious hygienic conditions, and this number is predicted to increase (UN-Habitat, 2003). There is a mental health crisis worldwide, with increased cases of anxiety, depression, and post-traumatic stress disorder (Brymer et al., 2019). Changes in climate and land use are affecting pathogen distribution, incidence, and behavior (de Souza and Weaver, 2024). The population growth rate also leads to higher global travel, an increased need for food production, and global trade, with higher use of antibiotics and pesticides, leading to antimicrobial resistance and environmental contamination, respectfully (Traore et al., 2023). Furthermore, our current relationship with animals goes beyond the need for food, and the human-animal bond is currently stronger than ever (Overgaauw et al., 2020). All those factors highlight how complex our current health problems can be—across species, transboundary, and multifactorial. To address those challenges, and to provide strategies and solutions that are sustainable and efficient, we need an approach that takes in a multisectoral and outreach approach, and One Health is our best option.

By promoting coordination, communication, collaboration, and capacity building across all sectors, a One Health approach can achieve broader success and the best health outcomes for people, animals, and plants in a complex and shared ecosystem. That is the only way we can then effectively transform the threat management approach, and expand beyond zoonoses and infectious diseases of pandemic potential, moving toward a more proactive global governance of risks (Elnaiem et al., 2023). However, to achieve that, the One Health approach needs to expand beyond the scientific world of publications and conferences. It is essential to include One Health in the organizational structure and curriculum of schools, colleges, and universities around the globe. The One Health community should be larger than traditional health professionals, they should include environmental scientists, social scientists, architects, engineers, and other professionals while connecting and engaging with community members to be involved in the work (Henley et al., 2021).

The One Health approach has become essential in our times, when we are facing a high number of major events affecting our health and our lives, including the COVID-19 pandemic (SARS-Cov-2), highly pathogenic avian influenza, climate change, food insecurity, social inequality, measles outbreak, and mental health issues. We need to embrace the old traditions of how we interact with nature, animals, ourselves, and our overall ecosystem. Bring back the ideal of connection that our long-gone native ancestors practiced while adapting it to our reality. Whether we recognize it or not, this ideal is at the center of the One Health approach. The collaborative aspect of One Health among all the sectors, stakeholders, and players involved becomes essential to promote the necessary broad outreach that will lead to sustainable solutions and most importantly, the difficult task of changing behavior on how we address disease prevention and promote health.

## Notes

Author Biography

**Elsio Wunder** is an Assistant Professor of One Health Approaches to Infectious Disease at the Department of Pathobiology and Veterinary Science, University of Connecticut. He is a trained veterinarian with more than 15 years of experience working in leptospirosis research laboratories, both in veterinary and public health settings. He received his PhD in Investigative Medicine from the Oswaldo Cruz Foundation, in Brazil, and has worked previously at the Yale School of Public Health. His research is focused on understanding the pathogenesis of leptospirosis, and, more specifically, identifying virulence factors in the spirochetal pathogen, which may in turn serve as the basis for novel approaches for diagnostics and vaccines. Beyond the pathogen, his research also focuses on the host/reservoir and the environment and is well aligned with the concept of the One Health approach. His laboratory collaborates with numerous researchers nationally and internationally to improve the field of research on this important neglected disease. Among other activities, he is a Visiting Researcher at the Oswaldo Cruz Foundation, Salvador, Brazil, and a Section Editor for Bacteriology in the journal PLoS Neglected Tropical Diseases. He currently teaches an undergraduate course specifically on the One Health topic, while integrating the One Health approach in all his courses at the University of Connecticut.
